# IgG1 and IgG4 antibodies sample initial structure dependent local conformational states and exhibit non-identical Fab dynamics

**DOI:** 10.1038/s41598-023-32067-9

**Published:** 2023-03-23

**Authors:** Ramakrishnan Natesan, Neeraj J. Agrawal

**Affiliations:** Process Development, Amgen Inc., 360 Binney St, Cambridge, MA 02141 USA

**Keywords:** Computational biophysics, Biophysics, Protein structure predictions

## Abstract

We have investigated the dynamics of two $\upgamma$-immunoglobulin molecules, IgG1 and IgG4, using long all atom molecular dynamics simulations. We first show that the de novo structures of IgG1 and IgG4 predicted using AlphaFold, with no interactions between the fragment crystallizable (Fc) domain and the antigen fragment binding domain (Fab), eventually relaxes to a state with persistent Fc–Fab interactions that mirrors experimentally resolved structures. We quantified the conformational space sampled by antibody trajectories spawned from six different initial structures and show that the individual trajectories only sample states bound by a local minimum and display very little mixing in their conformational states. Furthermore, the dynamics of the individual Fab domains are strongly dependent on the initial crystal structure and isotype. In all conditions, we observe non-identical dynamics between the Fab arms in an antibody. For a six-bead coarse grained model, we show that non-covalent Fc–Fab interactions can modulate the stiffnesses associated with Fc–Fab distances, angles, and dihedral angles by up to three orders of magnitude. Our results clearly illustrate the inherent complexities in studying antibody dynamics and highlight the need to include non-identical Fab dynamics as an inherent feature in computational models of therapeutic antibodies.

## Introduction

Monoclonal antibodies (mAbs) are large molecules of the immunoglobulin family. They constitute a key therapeutic modality due to their specificity, affinity, and their ability to bind to a wide range of cell surface receptor and soluble ligands^1,2^. Nearly all therapeutic mAbs are $\upgamma$-immunoglobulin (IgG) molecules comprised of four isotypes namely IgG1, IgG2, IgG3, and IgG4. IgG molecules are glycosylated heterodimers constituted of two heavy and two light chains. These polypeptide chains can be broadly divided into a variable region, that governs antigen binding, and a constant region, that governs effector function. The four IgG isotypes differ mainly in sequences contained in the variable region of their heavy chains. All IgG molecules can broadly be subdivided into two “fragment, antigen binding (Fab)” domains one “fragment, crystallizable (Fc)” domain^3,4^. Henceforth, we denote the Fab domains associated with heavy chains 1 and 2 as Fab1 and Fab2, respectively. It has been shown that antibodies are highly flexible molecules that can adopt extreme asymmetric conformations mainly due to the disordered hinge regions that connect a Fab domain to the Fc region^5,6^. Understanding the relation between mAb *“structure, dynamics, and function”* is of utmost importance for antibody (Ab) engineering, a key step in the antibody development pipeline^7,8^.

In a recent article^9^, we used long all-atom molecular dynamics (MD) simulations of the IgG1 b12 crystal structure (PDB ID: 1HZH^10^) from the RCSB protein data bank^11,12^ and demonstrated that its Fab1 and Fab2 regions exhibited non-identical dynamics despite containing identical sequences. We showed that the observed non-identical Fab dynamics is a result of persistent non-covalent interactions between the Fc and Fab regions. Saporiti and coworkers have also reported differential Fab dynamics in long all atom explicit solvent simulations of another IgG1 molecule adalimumab^13^. The presence of asymmetric Fab structures has also been verified in solution structures of full length IgG1 molecules. Rayner et al.^14^ have shown that the solution structures IgG1 6a and 12a molecules also possess stable asymmetric Fab arm arrangements identical to that observed for IgG1 b12^5,10^. Both X-ray and neutron diffraction studies showed that the asymmetric Fab structure of both molecules were stable under a range of salt concentrations and temperatures.

To better understand the phenomenon of asymmetric Fab arrangement and dynamics, in this article, we present a more in-depth analysis by extending our simulations to four other full length IgG crystal structures (PDB ID: 5DK3^15^, 6GFE^16^, 1IGT^17^, and 1IGY^18^), and to two immunoglobulin isotypes (IgG1 and IgG4). As an alternate approach, we also leveraged recent advances in deep learning methods for protein structure prediction^19–22^ to generate three dimensional structures of full length IgG1 and IgG4 antibodies which were then relaxed, equilibrated and used as the starting structures for MD simulations. Our study covering six different starting structures derived from experiments and machine learning methods was specifically designed to address two key questions: (i) is the non-identical Fab dynamics observed in our earlier study solely a feature of the 1HZH structure? (ii) do all crystal structures represent the same equilibrium state of an antibody and does the trajectories generated from different crystal structures sample similar conformational states?

The IgG1 and IgG4 molecules used in our study contain 1324 and 1318 residues, respectively, and both form 16 disulfide bonds between their cysteine residues. All mAbs used in this work have VK1 germline and VH3 germline sequences for the variable light and heavy chain regions. The structures in this report are named after their template PDB ID even though they differ in the sequence from their templates. Most IgG4 mAbs used as therapeutic antibodies contain a S228P modification to prevent Fab-arm exchange in denaturing conditions^23,24^ and is the most therapeutically relevant IgG4 molecule. In this manuscript, we refer this isoform with the S228P mutation as IgG4. More details about the simulations may be found in “Materials and methods”.

## Results and discussions

### Rapid relaxation of AlphaFold generated structures and onset of non-covalent interactions

**Figure 1:**
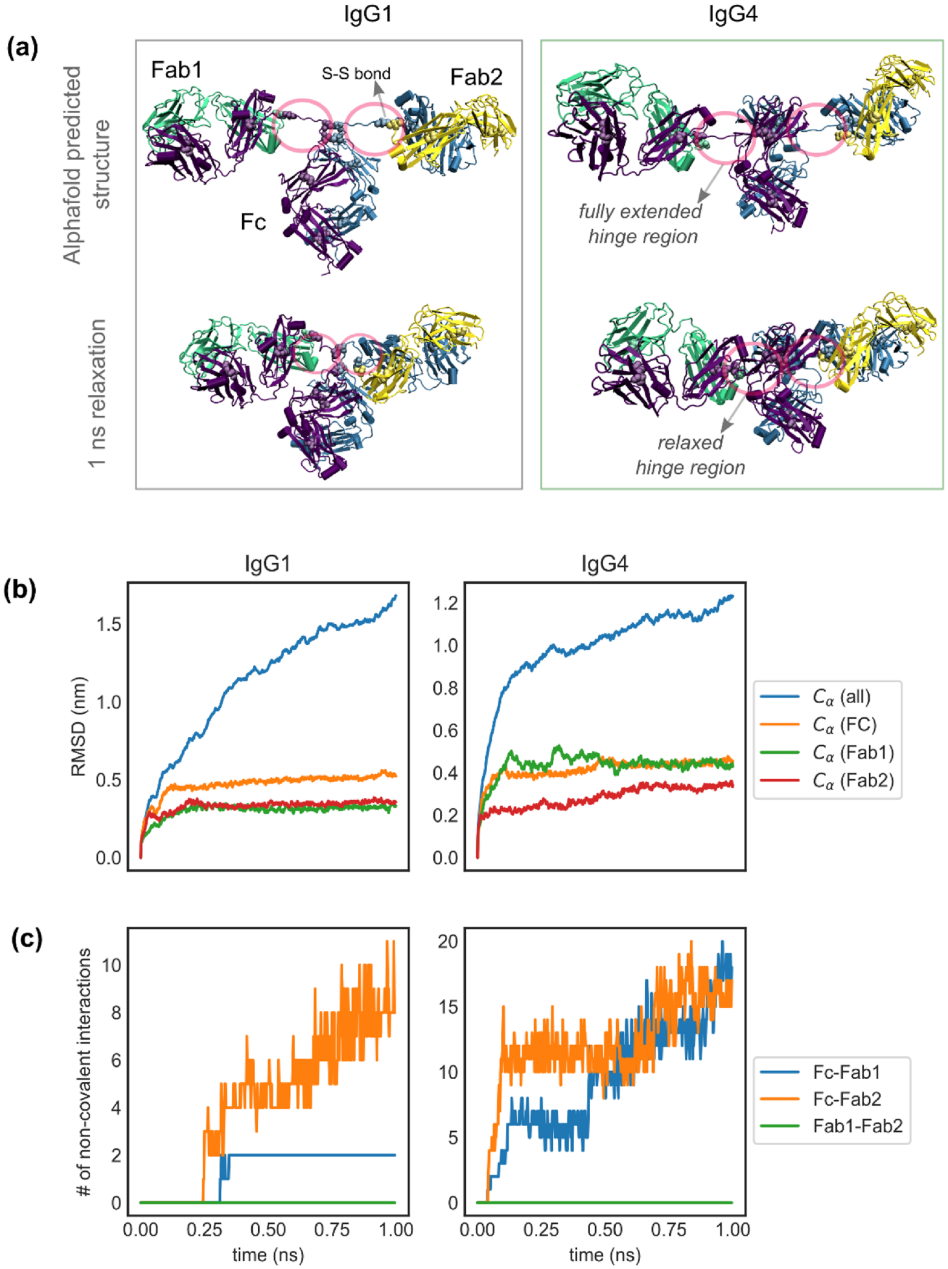
(**a**) Snapshots showing AlphaFold predicted structures of IgG1 and IgG4 antibodies and the corresponding conformations after 1 ns of molecular dynamics relaxation. The positions of cysteine residues forming disulfide bonds are displayed as Van der Waals beads and the spatial location of the hinge regions are marked by circles. (**b**) Time series of the root mean squared deviation (RMSD) in the alpha carbon (${C}_{\alpha })$ positions for the whole antibody (all), Fc, Fab1 and Fab2 domains. The individual domains undergo a minor rearrangement and stabilize with 0.1 ns, while the rmsd for the whole antibody shows a growing trend even past 1 ns. (**c**) Time series showing the number of non-covalent interactions between the Fc, Fab1 and Fab2 domains. Fc–Fab2 interactions are dominant in IgG1 while the Fc interacts with both Fabs in IgG4. Fab1–Fab2 interactions are non-existent in both molecules.

We generated de novo structures of the full length IgG1 and IgG4 sequences using the AlphaFold-multimer tool as described in the “Materials and methods” section. These structures, henceforth referred to as “AlphaFold structures”, for both IgG1 and IgG4 closely resemble the symmetric-**T** conformation of antibodies (Fig. 1a) and did not show any inter-domain interactions between the Fab1, Fab2, and Fc domains. When further relaxed, the AlphaFold structures showed rapid equilibration to** Y** like conformations within a short span of 1 ns (Fig. 1a). The timeseries of the RMSDs for the Fab1, Fab2, and Fc domains show that the local structures of the individual domains only undergo minor rearrangements and stabilize within 0.1 ns (Fig. 1b). However, the observed trend in the RMSD for the whole antibody indicates that the AlphaFold structures are far separated from their respective global minima and would require much longer simulations to relax. The relaxation to the closest minima is mainly driven by the dynamics of the initially extended hinge regions (Fig. 1a,b). As the hinge regions relax, the conformational states of the Fc and Fab domains start to overlap and as result they interact with each other via persistent non-covalent bonds (Fig. 1c). Our analysis of these interactions in both IgG1 and IgG4 antibodies shows that the Fc region interacts primarily with Fab1 and Fab2 domains and to an extent with the hinges. We did not observe any inter-Fab interactions in our analysis for both molecules. It should be noted that the de novo generated IgG1 structure also shows dominant Fc–Fab2 interactions, as reported in our previous study. These results establish that the non-covalent Fc–Fab interactions reported in our earlier study of IgG1 b12 structure 1HZH^9^ is not an artifact of the crystal structure but rather a feature of the antibody. Furthermore, it also clearly evidences that the deep learning approaches for protein structure prediction, e.g., AlphaFold^19^, EMBER2^20^, RoseTTAFold^21^, can only predict the local domain structures of large molecules, such as an antibody, to a very high accuracy. Conventional biophysics-based methods should be used in conjunction with such machine learning based approaches to ascertain the closest minimum structure that are essential for functional modeling.

N-glycosylation is a major post translational modification found in all antibodies and is known to play a key role in determining antibody safety and efficacy profiles. Nearly all antibodies contain N-linked glycans on their heavy chains in the Fc region (Asn-298 for IgG1 and Asn-290 for IgG4)^25^. In long all atom molecular dynamics simulations, the presence of glycans and more importantly those containing fucose were shown to impose constraints on the three dimensional conformations of all domains in the mAb^13^. To study more realistic models of antibodies we used the relaxed AlphaFold structures to generate N-glycosylated IgG1 and IgG4 antibodies by tethering an A2G0 glycan^26,27^, the simplest of the biantennary glycans, at each of the N-glycosylation sites. The glycosylated structures were first relaxed in explicit solvent for 30 ns and the dynamics of the equilibrated structure were then studied using long implicit solvent simulations.

**Figure 2:**
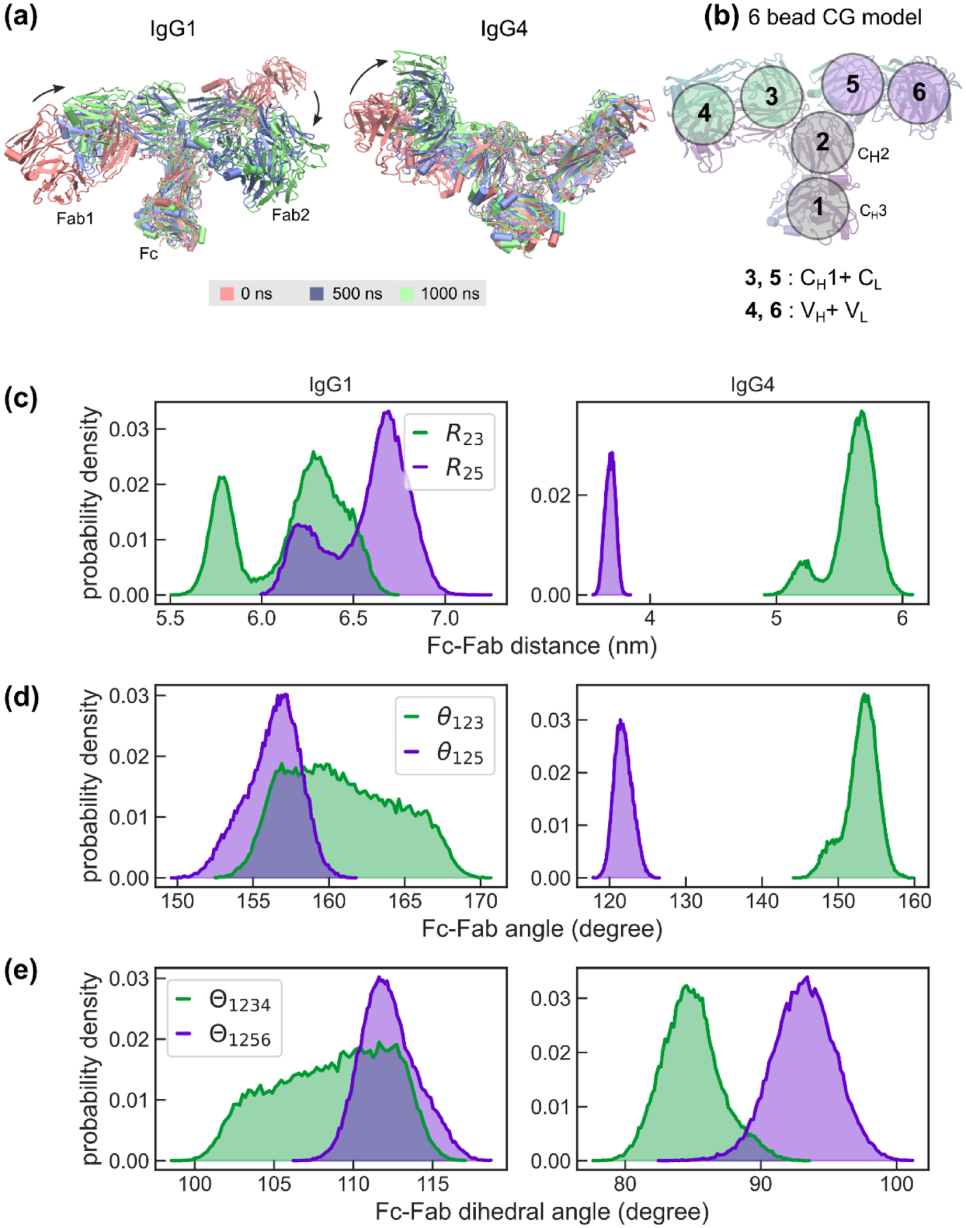
Dynamics of glycosylated IgG1 and IgG4 molecules generated using the AlphaFold predicted structures showing differential dynamics of the Fab arms. Data for the presented analysis were generated from 1000 ns implicit solvent simulations. (**a**) Comparison of antibody conformations at 0, 500, and 1000 ns showing large relaxation dynamics in the Fab domains for both IgG1 and IgG4 molecules. The arrows are a guide to the eye to mark the predominant directions of motion. (**b**) An illustration of the six-bead model used to quantify interdomain dynamics, with the antibody domains associated with each of the beads marked. (**c**–**e**) Probability densities of Fc–Fab distances (${R}_{23}$ and ${R}_{25}$), Fc–Fab angles (${\theta }_{123}$ and ${\theta }_{125}$), and Fc–Fab dihedral angles (${\Theta }_{1234}$ and ${\Theta }_{1256}$). Data for IgG1 and IgG4 molecules are shown in the left and right panels, respectively.

Results from 1000 ns long individual implicit solvent simulations of glycosylated IgG1 and IgG4 structure, initially generated using AlphaFold, are shown in Fig. 2. The superposition of antibody conformations at 0, 500, and 1000 ns time points in Fig. 2a clearly highlights large relaxations in the Fab domains, within the first 500 ns. The solid arrows in Fig. 2a that mark the directions of the dominant relaxation dynamics are shown as a guide to the eye. We further analyzed the long dynamics of IgG1 and IgG4 antibodies using a six-bead coarse-grained model, introduced in our previous work^9^, and also shown in Fig. 2b. The six-bead model allows us to effectively quantify differential inter-domain fluctuations of antibodies in terms of their Fc–Fab distances (${\text{R}}_{23}$ and ${\text{R}}_{25}$)*,* Fc–Fab angles (${\uptheta }_{123}$ and ${\uptheta }_{125}$)*,* and Fc–Fab dihedral angles (${\Theta }_{1234}$ and ${\Theta }_{1256}$)*.* ${R}_{23}$ and ${R}_{25}$ (see Fig. 2c) display non-identical equilibrium distributions, for both IgG1 and IgG4, indicating that one of the Fab arms in the antibody fluctuates more freely compared to the other. For instance, ${R}_{25}$ that quantifies Fab2 dynamics in our simulations is dominant in IgG1 but is severely constrained in IgG4. Similarly, differential dynamics in other antibody degrees of freedom can be readily seen in the distributions of Fc–Fab angles ${\uptheta }_{123}$ and ${\uptheta }_{125}$ (Fig. 2d) and Fc–Fab dihedral angles ${\Theta }_{1234}$ and ${\Theta }_{1256}$ (Fig. 2e). It should be noted that the dynamics of a Fab arm is strongly dependent on its non-covalent interactions with the Fc-region and either of the Fab arms can exhibit dominant dynamics with an equal probability.

### Comparison of Ab dynamics spawned from six different initial structures

**Figure 3:**
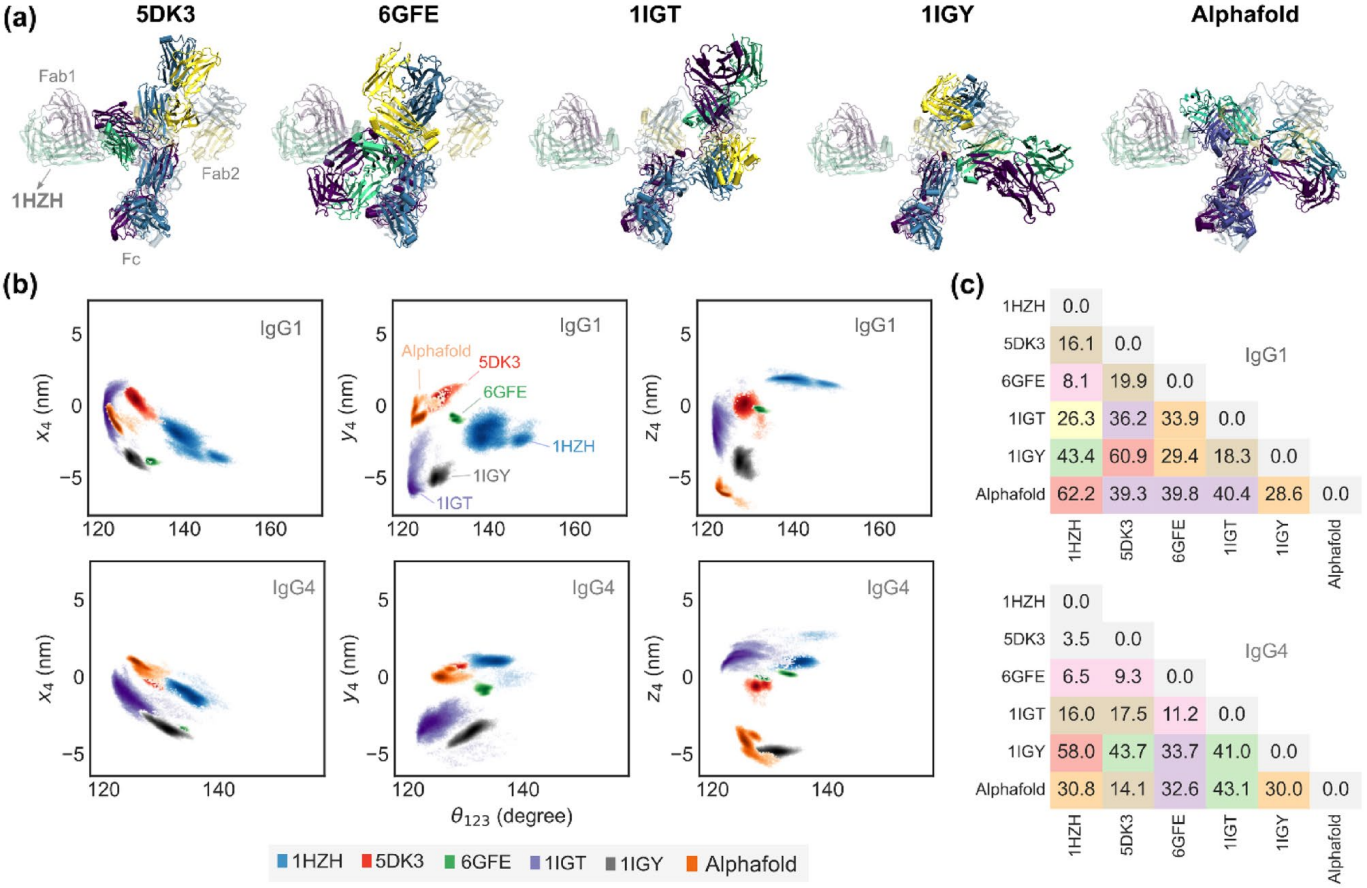
(**a**) Snapshots of IgG1 molecules generated by homology modeling of 1HZH, 5DK3, 6GFE, 1IGT, 1IGY crystal structures, all aligned to the Fc region of 1HZH. The reference 1HZH structure is shown in the background for comparison. The conformation of the AlphaFold structure post 1000 ns of relaxation is also shown. (**b**) Spatial occupancy density plots for each structure, computed from two independent 500 ns trajectories, show ${x}_{4}$, ${y}_{4}$, and ${z}_{4}$, the x, y, and z positions of bead 4, vs ${\theta }_{123}$. The point clouds associated with each group was constructed from around 5e4 observations. The conformational states accessed by each of the structures show minimal overlap in Cartesian space, pointing to the presence of locally minimum states in both IgG1 and IgG4 structures. (**c**) The spatial separation between the conformational states accessed by the various structures is quantified by the Kantorovich–Wasserstein distance metric, ${d}_{KW}$. For reference, ${d}_{KW}=0$ for two identical clouds of points, and ${d}_{KW}=2.6$ for two normally distributed clouds of points centered around (0,0,0) and (2,0,0), respectively.

Despite starting from the AlphaFold initial structure with zero inter-domain interactions, upon sufficient relaxation an antibody settles into conformational states with dominant non-covalent Fc–Fab interactions. Such inter-domain interactions have also been observed in various crystal structures of full-length antibodies each of which display a unique conformational state and non-covalent interaction profile. As a case in point, we used homology modeling to generate five different structures of IgG1 and IgG4 antibodies corresponding to five full-length crystal structures of IgG molecules available on the RSCB protein data bank^11,12^ with PDB IDs: 1HZH, 5DK3, 6GFE, 1IGT, and 1IGY. Details of homology modeling are presented in the “Materials and methods”. An overlay of the homology generated structures, with the Fc domain of 1HZH as the reference, is shown in Fig. 3a for IgG1 and SI Fig. S1 for IgG4. The AlphaFold generated structure relaxed for 1000 ns is also shown alongside for comparison. We specifically chose the 1HZH crystal structure as the reference to directly compare our results with our earlier study. It can be clearly seen that each of the structure is distinctly different from the other with very minimal overlaps in their Fab domains. Even the structure of antibodies generated in a crystal structure independent manner, using AlphaFold, relaxes to a distinct conformation that has minimal overlap with previously reported experimental structures. This raises the question if these antibody conformations represent true global minima or are more representative of individual local minima. We next set out to determine if these structures truly represent the equilibrium conformational states of an antibody by analyzing the overlap in their trajectories using long all atom implicit solvent simulations. All homology modeling derived structures were first prepared and equilibrated for 10 ns in explicit solvent conditions (see “Materials and methods”) and the fully relaxed structures were used as the starting structures for the long implicit solvent simulations.

We generated at least two independent 500 ns long trajectories, for both IgG1 and IgG4, using the homology modeling generated conformations as the initial structure and analyzed their dynamics using the six-bead model, with the Fc-region of the 1HZH starting structure as the reference. All trajectories showed dominant but diverse non-covalent interactions between the Fc region and at least one of the Fab domains (see SI Figs. S2 and S3), highlighting the role of Fc–Fab interactions in governing antibody conformations and dynamics.

### Fab domains in trajectories started from different initial structures show nearly zero overlap in their conformational states

The spatial positions explored by the antibody in our long simulations initiated with six different starting states display very minimal overlap in three-dimensional (3D) space indicating nearly complete segregation of the conformational states explored by each trajectory. This effect is more pronounced for beads 4 and 6 that represent the variable domains of Fab1 and Fab2, respectively. Figure 3b shows the spatial map of bead 4 in terms of its spatial positions ${x}_{4}$, ${y}_{4}$, and ${z}_{4}$ against the corresponding Fc–Fab angle ${\theta }_{123}$ for both IgG1 and IgG4. The corresponding projections in the Cartesian space for all Fab domains is displayed in SI Figs. S4 and S5. The occupancy map clearly shows strong localization of the trajectories and very nearly zero overlap of their conformational states. For instance, the states occupied by the 1HZH and 6GFE structures in IgG1 show some degree of overlap along the $z$-coordinates but are far apart in the $x$ and $y$ directions, implying that their trajectories explore mutually exclusive conformational states in 3D-space resulting in nearly zero transition probability between them. In both IgG1 and IgG4, desegregation of the conformational states is more pronounced for the variable domains (beads 4 and 6) compared to the constant Fab domain (beads 3 and 5), see SI Figs. S4 and S5. The spatial separation between the conformational states can be better understood in terms of the Kantorovich–Wasserstein distance metric (${d}_{KW}$) that quantifies the separation between two cloud of points in terms of a single non-dimensional scalar^28^. For example, ${d}_{KW}=0$ for two identical cloud of points and ${d}_{KW}=2.6$ for two normally distributed, marginally overlapping, point clouds with their respective peaks at (0,0,0) and (2,0,0). Estimates for ${d}_{KW}$, computed as described in the “Materials and methods”, for bead 4 is shown in Fig. 3c. Our calculations of ${d}_{KW}$ for the IgG1 occupancy map shows that the 1HZH and 6GFE structures share closely separated conformational states with ${d}_{KW}=8.1$, while 5DK3 and 1IGY structures have far-separated conformational states with ${d}_{KW}=60.9$. On the other hand, for IgG4 molecules the conformational states of 1HZH and 5DK3 are the closest with ${d}_{KW}=3.5$, and that of 1IGY and 1IGT are the farthest with ${d}_{KW}=58$.0. It should also be noted that the conformational states accessed by the AlphaFold structure is nearly equidistant from all crystal structures for IgG1.

**Figure 4:**
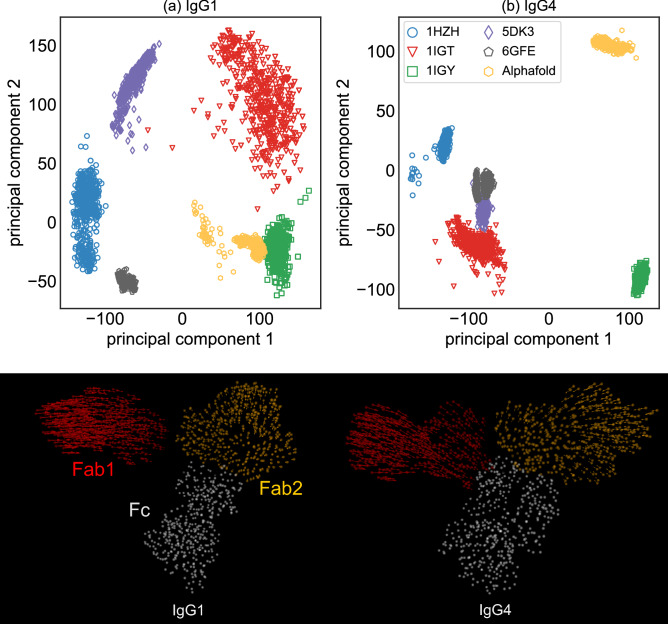
Projections of six independent trajectories (spawned from five different crystal structures + from the relaxed structure predicted by Alphafold), for (**a**) IgG1 and (**b**) IgG4, along their first two principal directions. Principal Component (PC) analysis was performed using the positions of all alpha carbon atoms over all trajectories. The conformational states accessed by each structure again displays minimal overlap in the PC space. The images below display the first principal direction for the 1HZH structure. The length of the arrows at each alpha carbon (shown as spheres) is directly proportional to the atom’s contributions to the principal motion. Our analysis shows that the first principal motion is more concentrated on the Fab1 domain, that shows zero non-covalent interactions with the Fc region.

We next studied the essential dynamics^29^ for each IgG isotype by performing a Principal Component Analysis (PCA) by combining six sets of trajectories (each with at least two replicates) for each isotype, as described in the “Materials and methods”. In our analysis, we only used the positions of the ${C}_{\alpha }$ atoms and all trajectories were aligned to the Fc region of a single frame in the 1HZH-spawned trajectories. The projections of each trajectory in the joint principal component (PC) space were computed and plotted as in Fig. 4a,b—here we display the projection in the PC plane defined by the first two principal vectors that account for nearly 91% of the total variance. As expected, replicate trajectories spawned from the same crystal structure clustered together but showed nearly zero overlap with those spawned from a different structure. Similar results were also observed in other PC planes, see SI Figs. S6, S7. Furthermore, our analysis also showed that the first six principal modes were dominated entirely by the motions of the unrestrained Fab1 domain (Fig. 4). Contributions to the first six PC vectors from the dynamics of the Fab2 domain, that was previously shown to exhibit restrained motion due to its non-covalent interactions, were minimal. These results strongly support our earlier findings and clearly indicate that the non-covalent Fc–Fab hydrogen bonds are indeed highly persistent and define a large energy barrier leading to non-identical Fab dynamics.

**Figure 5:**
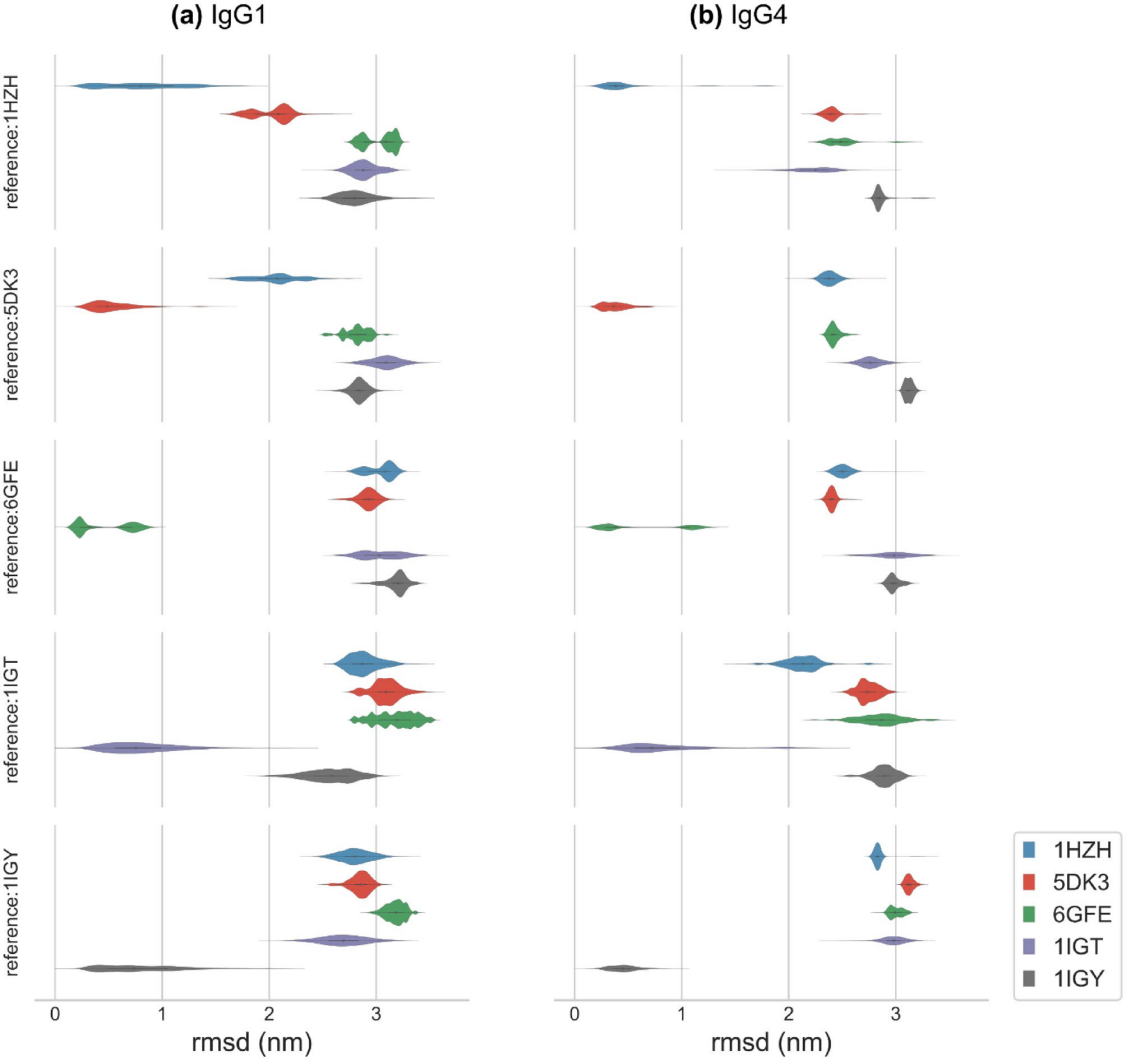
Violin plots showing the RMSD distributions for (**a**) IgG1 and (**b**) IgG4 trajectories spawned from the five full length antibody structures discussed in Fig. 3. A typical 500 ns trajectory in our studies contained 25,000 frames and every system contained two independent trajectories. Each frame in the trajectory was aligned to 25 conformations, each 20 ns apart, in a reference trajectory and RMSD was computed for each alignment. Thus, every distribution in the plot was computed from 1.25 million estimates. RMSD for cross alignment in which the trajectory structure differs from the reference is significantly higher than RMSD for self-alignment indicating that trajectories spawned from a crystal structure only samples conformational states around its initial conformation.

To rule out the possibility that the observed segregation of conformational states is an artifact of the chosen reference structure we aligned all the trajectories, spawned from the six different initial structures to 25 different antibody structures in a reference trajectory and computed the corresponding RMSD scores for the alignment—the 25 structures corresponds to antibody conformations 20 ns apart in a 500 ns trajectory. We expected to see significant reduction in the RMSD score if a trajectory overlaps with one or more of the 25 reference structures. Our analysis for IgG1 and IgG4 for the five different reference structures is shown in Fig. 5. For each reference trajectory (1HZH, 5DK3, 6GFE, 1IGT, and 1IGY) it may be noted that the RMSD score is minimum for the trajectory spawned from the same crystal structure. Consistently, all the other crystal structures show significantly higher RMSD scores with a very minimal overlap, except for 1HZH and 5DK3 in the case of IgG1, and 1IGY and 1IGT for both IgG1 and IgG4. It should also be noted that a lower value of ${d}_{KW}$ do not translate to a lower RMSD score, for instance 1HZH and 6GFE for IgG1 in Fig. 3c. This is not surprising since distance metrics such as the Kantorovich–Wasserstein distance (${d}_{KW}$) are not orientation-aware, while the RMSD is. These results clearly show that antibodies spawned from different initial structures sample conformational states around their starting conformation and do not readily transition across states, suggesting that each of the antibody conformations are trapped in a local minimum defined primarily by its non-covalent Fc–Fab interactions. Biased sampling techniques should be employed to understand the nature of these local minima and quantify the transition between the various states. We will pursue this line of work in a follow up article. In the next section, we will explore the dependence of Fab arm dynamics on the initial structure of the antibody.

### Fab arm specific non-covalent interactions strongly influence Fab dynamics in all simulated antibody trajectories

**Figure 6:**
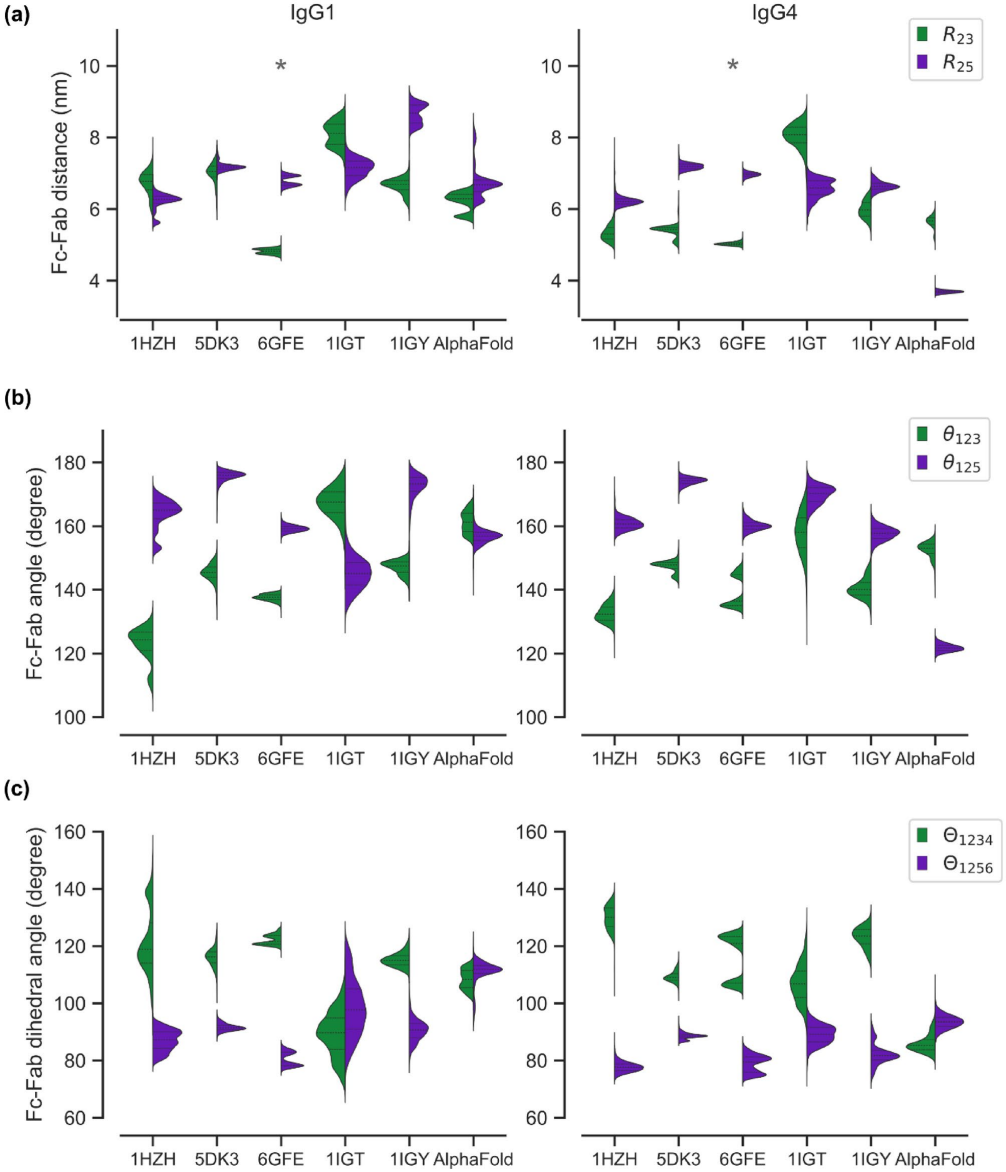
Comparison IgG1 and IgG4 dynamics for six different starting structures. In addition to the AlphaFold generated structure, five other initial conformations of the antibodies were generated from crystal structures (1HZH, 1IGT, 1IGY, 5DK3 and 6GFE) using homology modeling. Data shown correspond to duplicate implicit solvent simulations run for at least 500 ns. The violin plots in panels (**a**)–(**c**) show the distributions of Fc–Fab distances (${R}_{23}$ and ${R}_{25}$), Fc–Fab angles (${\theta }_{123}$ and ${\theta }_{125}$), and Fc–Fab dihedral angles (${\Theta }_{1234}$ and ${\Theta }_{1256}$), respectively, computed for the 6-bead model show in Fig. 2b. The asterisk marks the 6GFE structure that shows nearly identical joint distributions for IgG1 and IgG4.

We extended our analysis presented in Fig. 2 to quantify the effect of an antibody’s initial structure on the dynamics of its Fab domains. Equilibrium fluctuations in the measures ${R}_{23}$ and ${R}_{25}$, ${\theta }_{123}$ and ${\theta }_{125}$, and ${\Theta }_{1234}$ and ${\Theta }_{1256}$ are displayed in Fig. 6a–c, respectively, for both IgG1 and IgG4. The corresponding number of non-covalent interactions between the Fc region and the Fab1 and Fab2 arms for all the trajectories are shown in SI Figs. S2 and S3. It should be noted that the multimodal distributions observed in Fig. 6 are not truly multimodal but represent data averaged over multiple ensembles. The ensemble specific distributions are displayed in SI Figs. S8–S13.

It may first be noted that the equilibrium distributions for both Fab arms are strongly dependent on the initial structure. For instance, the joint distribution of ${R}_{23}$, ${\theta }_{123}$, and ${\Theta }_{1234}$ for trajectories spawned from the 1HZH structure differs from those generated from other crystal structures. The dependence on initial structure is further reflected in the number of Fc–Fab interactions quantified in SI Fig. S3. In the case of IgG1, one of the Fab arms showed more dominant interactions with the Fc region. For example, Fc–Fab1 interactions were dominant in trajectories initiated from 1IGY, 6GFE and the AlphaFold structures, while Fc–Fab2 interactions are dominant in trajectories spawned from 1HZH and 5DK3 structures. Unlike for IgG1, both Fab arms in IgG4 show strong non-zero interactions with the Fc domain. In our long simulations, we observed the time-averaged number of non-covalent interactions to span a vast range from 2 hydrogen bonds (for 1IGT in IgG1) to 120 hydrogen bonds (for AlphaFold structure in IgG4). If we assume that the local minimum associated with each crystal structure is entirely defined by non-covalent interactions, we can estimate the depth of these local minima to be in the range ~ 2–120 kcal/mol—we take the energy of a hydrogen bond to be 1 kcal/mol^30^. These findings taken together clearly illustrate that Fab arm dynamics in an antibody is strongly dependent on its initial structure.

Secondly, we observed non identical Fab arm dynamics in all antibody trajectories spawned from the six different structures. Non identical Fab dynamics is marked either by a shift in the equilibrium values or by a change in the distribution profile or both. As reported in our earlier study^9^, we observed strong Fc–Fab2 non-covalent interactions in 1HZH trajectories leading to restricted Fab2 arm dynamics. Similarly, all simulated antibody trajectories spawned from the six structures displayed some degree of variation between individual Fab arm dynamics, suggesting that non-identical Fab dynamics is inherent to antibody motion.

Finally, the dynamics of the Fab arms not only depend on the initial crystal structure but also on the molecule isotype. A comparison of IgG1 and IgG4 dynamics started from the same initial structure (see Fig. 6) showed entirely different distributions, non-covalent interaction profiles, and differential Fab dynamics thus highlighting the role of molecular isotype on antibody dynamics. In all our simulations for IgG1 and IgG4 only the trajectories for the 6GFE structure showed identical Fab dynamics and non-covalent interactions across both isotypes (Fig. 6, and SI Fig. S3). Note that, 6GFE is the most constrained of all IgG structures with a $\uplambda$ conformation that allows both IgG1 and IgG4 to form large number of non-covalent interactions that dominate their dynamics.

### Stiffness parameters estimated for the six bead model are strongly influenced by Fc–Fab interactions

Coarse grained models of antibodies are regularly used in a number of contexts, such as predicting Ab viscosity^31^, study Ab self-association^32,33^ and to identify physical instabilities at higher Ab concentrations^34^. For a full list of applications see^35^. Results from our extensive analyses clearly illustrate that the underlying assumption of these models that both Fab arms in an antibody exhibit identical dynamics is very simplistic. To demonstrate how non-covalent interactions can modulate the parameters for a coarse-grained model, we computed a few of the stiffnesses parameters in our six-bead model that involves both the Fc and Fab regions. The corresponding interaction potentials may be represented by the harmonic terms given below:

(i) ${{\varvec{E}}}_{\mathbf{distance}}=\frac{{{\varvec{k}}}_{23}}{2}{\left({{\varvec{R}}}_{23} -\overline{{{\varvec{R}} }_{23}}\right)}^{2}+\frac{{{\varvec{k}}}_{25}}{2}{\left({{\varvec{R}}}_{25}-\overline{{{\varvec{R}} }_{25}}\right)}^{2}$ with ${k}_{23}$ and ${k}_{25}$ being the spring constants associated with the Fc–Fab distance fluctuations in Fig. 6a,
(ii) ${{\varvec{E}}}_{\mathbf{angle}}=\frac{{{\varvec{k}}}_{123}}{2}{\left({{\varvec{\uptheta}}}_{123}-\overline{{{\varvec{\uptheta}} }_{123}}\right)}^{2}+\frac{{{\varvec{k}}}_{125}}{2}{\left({{\varvec{\uptheta}}}_{125}-\overline{{{\varvec{\uptheta}} }_{125}}\right)}^{2}$ with ${k}_{123}$ and ${k}_{125}$ being the stiffnesses associated the Fc–Fab angle fluctuations in Fig. 6b, and
(iii) ${{\varvec{E}}}_{\mathbf{dihedral}}=\frac{{{\varvec{k}}}_{1234}}{2}{\left({{\varvec{\Theta}}}_{1234}-\overline{{{\varvec{\Theta}} }_{1234}}\right)}^{2}+\frac{{{\varvec{k}}}_{1256}}{2}{\left({{\varvec{\Theta}}}_{1256}-\overline{{{\varvec{\Theta}} }_{1256}}\right)}^{2}$ with ${k}_{1234}$ and ${k}_{1256}$ being the stiffnesses associated the Fc–Fab dihedral angle fluctuations in Fig. 6c.

**Figure 7:**
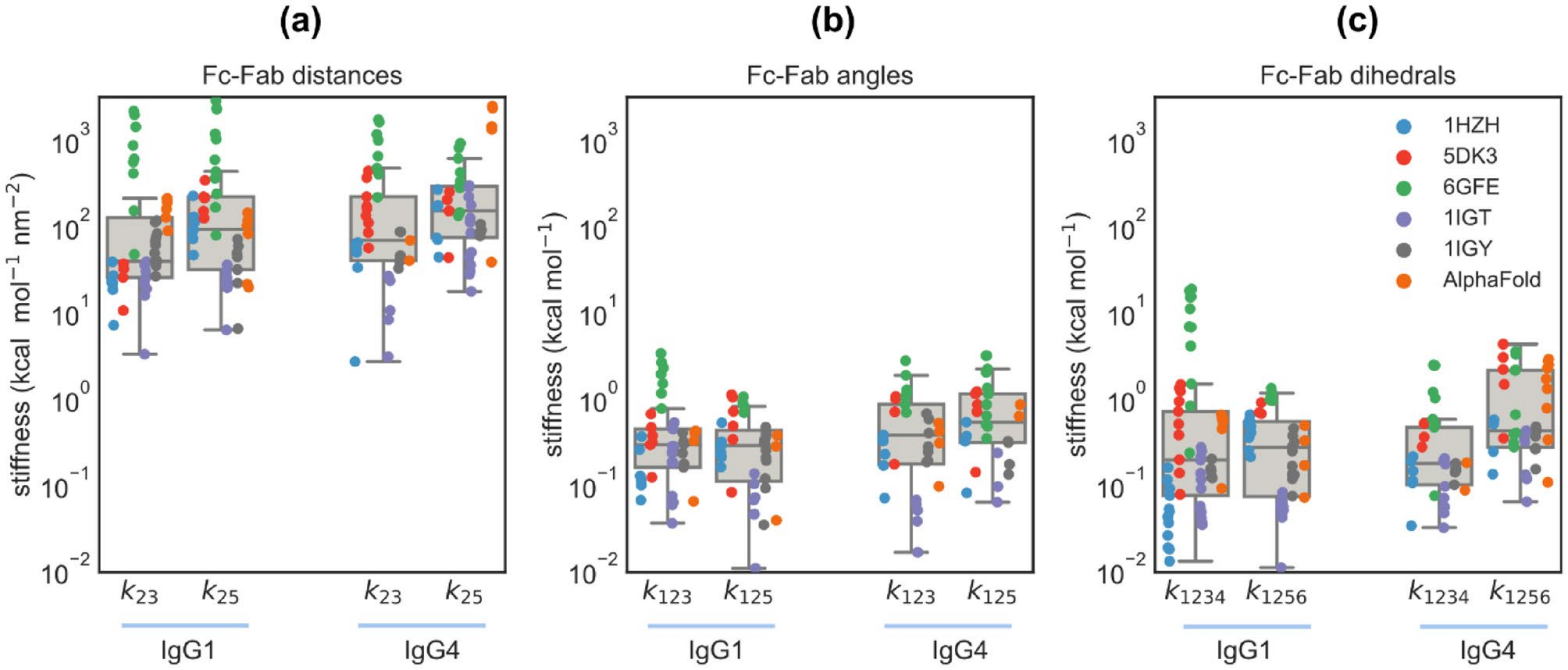
Estimates for stiffnesses parameters associated with (**a**) Fc–Fab distances (${k}_{23}$ and ${k}_{25}$) and (**b**) Fc–Fab angles (${k}_{123}$ and ${k}_{125}$) and (**c**) Fc–Fab dihedral angles (${k}_{1234}$ and ${k}_{1256}$), grouped by antibody isotype, showing a strong dependence on the initial structure and a weak dependence on the isotype. The scatter dots in each group correspond to stiffness estimates obtained using an unsupervised Gaussian mixture model for trajectories spawned from the marked initial structure, while the boxplot quantifies the spread of data over all initial structures.

We applied an unsupervised Gaussian mixture model^36^ to the distributions in Fig. 6 and estimated the various spring constants and angle stiffness described above. This machine learning model was set up to automatically determine the optimal number of Gaussians required to best fit the distribution. The estimated covariance ${\sigma }^{2}$ for each component Gaussian was converted to a spring constant $k$ using the expression $k={K}_{B}T/{\sigma }^{2}$, with ${K}_{B}$ being the Boltzmann constant and $T$ being the absolute temperature, taken to be 300 K. The estimates for the various stiffnesses determined from trajectories spawned from all six different structures, for both IgG1 and IgG4 antibodies, are displayed in Fig. 7. For both IgG1 and IgG4, our estimates for ${k}_{23}$ and ${k}_{25}$ are in the range 2–2900 kcal mol^−1^ nm^−2^ and are proportional to the number of non-covalent Fc–Fab interactions in the structure. For example, it may be seen that ${k}_{23}$ for 1IGT (with a maximum of 10 Fc–Fab interaction in both isotypes) is consistently lower compared to that for 6GFE (with roughly 50–65 bonds), see Figs. S2 and S3. Similarly, ${k}_{25}$ for the AlphaFold trajectories displays a lower range for IgG1 (~ 30 bonds) compared to IgG4 (~ 60 bonds). We observed a similar dependence in our estimates for ${k}_{123}$ and ${k}_{125}$ with values in the range 0.01–3.3 kcal mol^−1^, and ${k}_{1234}$ and ${k}_{1256}$ with values between 0.01–18 kcal mol^−1^. The three orders of magnitude variation in the estimates for the coarse-grained model parameters clearly highlights the importance of accounting for non-covalent interactions in developing coarse grained models for antibodies since variations across such a vast range can significantly influence the physical properties measured from the model. We also note that it is not straightforward to quantify complex Fc–Fab interactions using the simple harmonic potentials given above. It would be a challenge particularly in quantifying the equilibrium values $\overline{{R }_{ij}}$, $\overline{{\uptheta }_{ijk}}$, and $\overline{{\Theta }_{ijkl}}$ shown above. This can be overcome by utilizing advanced methods such as torchMD^37^ that performs molecular simulations by first learning the optimal potentials from the trajectories using a deep neural network architecture.

## Conclusions

We set out to determine (i) if the non-identical Fab dynamics previously reported in simulations of full-length IgG1 is an artifact of the starting crystal structure and (ii) if the experimentally resolved full length crystal structures of IgG molecules are sampled from the same canonical ensemble. We addressed these questions using long all atom MD simulations of full-length antibodies (> 500 ns in duplicates) starting from five different starting conformations generated from the five different crystal structures of full-length IgG molecules. All simulations were performed in implicit solvent conditions using the AMBER force field in OpenMM. We also generated de novo structures of full length IgG1 and IgG4 molecules using AlphaFold multimer and studied the relaxation dynamics of the computationally determined structures. Our results clearly establish (i) irrespective of the initial structure, non-covalent Fc–Fab interactions, and the resulting non-identical Fab dynamics, is a ubiquitous feature of antibody dynamics and (ii) trajectories spawned from different starting conformations have completely segregated conformational states suggesting the experimentally determined IgG structures do not represent a canonical ensemble but rather a statistical ensemble with its own local minimum.

We have discussed the implications of our results particularly in the context of developing coarse grained models, for instance to estimate Ab viscosity, study Ab self-association in crowded environments, and predict physical and chemical instabilities of Ab solutions. Conventionally, these models treat both Fab arms in an antibody to exhibit identical dynamics and are parameterized as such. We have demonstrated that non-covalent Fc–Fab interactions can modulate the stiffness parameters for a coarse-grained model up to three orders of magnitude suggesting that realistic coarse-grained models of antibodies should also account for non-identical Fab dynamics.

A handful of experimentally determined crystal and solution structures of full length IgG1 antibodies have clearly established the presence of asymmetry in the arrangement of Fab arms^5,10,14^. It has been postulated that the Fab conformations are modulated to allow the Fc region to adopt conformations suitable for complement binding and promote antibody effector functions. The presence of non-covalent interactions between the Fc and Fab regions and the associated local minima reported in our article is supported by the Levinthal postulate^38^ that states “more often, than not, large polypeptide chains settle into locally minimum energy states rather than seeking the state that minimizes the Gibb’s free energy”. Whether antibodies in these locally minimum conformational states possess the same biological function and efficacy in vivo remains an open question. Though the trajectories generated in our study are sufficiently long for atomistic molecular simulations, they are not sufficiently long to fully understand the stability of the local minima. Enhanced sampling techniques should be used to fully understand the complex free energy landscape of therapeutic antibodies. In summary, our work highlights the inherent complexities in antibody dynamics and the importance of developing a better understanding of *“antibody structure-dynamics-function axis”* for efficient antibody engineering.

## Materials and methods

### Homology modeling to generate structures of IgG1 and IgG4 antibodies

We used *BIOVIA Discovery Studio 2020* to generate the three-dimensional structures of full length IgG1 and IgG4 molecules via homology modeling. IgG1 and IgG4 sequences were constructed by merging VK1/VH3 germline variable region sequence with the constant region sequences. We used the crystal structures (PDB IDs: 1HZH, 1IGT, 1IGY, 5DK3, and 6GFE) downloaded from the RCSB protein data bank as the template sequence. For each template, five different initial models were generated using the "Model Full length antibody" protocol in Discovery Studio v20 (Modeler v 9.22) and the models were ranked per their *probability density functions *(PDF) total energy^39^. The model with the lowest PDF total energy was selected for MD simulations since the lower PDF energy indicates that the model satisfies the homology restraints better. The glycosylation form on each of these modeled structures was adjusted to A2G0. The homology generated structures were relaxed and used as the starting conformation for further studies.

### Structure preparation and relaxation using Ambertools

AlphaFold generated de novo structures of IgG1 and IgG4 were prepared for MD simulations using *tleap*^40^. Briefly, we first determined the correct protonation states of the histidine residues using the H++ webserver. We used *pdb4amber* to fix the correct histidine residues, mutate all cysteines to CYX, and the glycan-linked asparagine residues to NLN. The missing heavy atoms and hydrogens, disulfide bonds and glycoprotein linkages were all added using *tleap*. The completed IgG1 and IgG4 glycoproteins each contained 20,502 and 20,409 atoms, and had charges of + 20e and + 12e, respectively. For explicit solvent simulations, the protein was first neutralized using Na^+^/Cl^−^ ions and were solvated in a rectangular periodic TIP3P water box, with 150 mM NaCl. The box dimensions were automatically computed to have at least 13A padding in each direction. The number of atoms for the explicit solvent system varied with the structure—the smallest system (1HZH) contained 979,668 atoms while the largest system (1IGY) contained 1,673,940 atoms.

For both the explicit and implicit solvent models, we minimized the prepared structures for 2000 cycles using conjugate gradient descent, with a non-bonded cutoff of 7A. The minimized structures were then relaxed via Langevin dynamics (*ntt* = *3, gamma_ln* = *1.0*) at 300 K with a 1 fs timestep, for 1 ns. Both these steps were performed using *sanders*^40^. The relaxed structures were then used for OpenMM simulations as described below.

### Molecular dynamics simulations

We performed all molecular dynamics simulations presented in this article with the open source tool *OpenMM*^41^ using AMBER *ff19SB*^42^ force field for proteins, and the *GLYCAM_06j-1* force field carbohydrates and glycopeptides^43^. All simulations were run in an NPT ensemble, with T = 300 K and P = 1 atmosphere. Covalent bonds involving hydrogens were constrained allowing us to use a timestep *dt* = 2 fs. The temperature and pressure were regulated using a Monte Carlo Barostat for which we chose a friction coupling of 1 ps^−1^, barostat interval of 25*dt, and a constraint tolerance of 1e−6. Explicit solvent simulations were performed in a rectangular periodic box (as described in structure preparation) by setting the water to be rigid. Non bonded interactions were evaluated using Particle Mesh Ewald with a non-bonded cutoff of 8A. For implicit solvent simulations periodic boundary conditions were removed and we used the Hawkins-Cramer-Truhlar GBSA model^44^, with salt concentration of 150 mM. In all our simulations the initial structure was first minimized, then equilibrated for 1 ns, followed by production runs. Typical runs of a N-glycosylated IgG1, with 20,502 atoms, on a single NVIDIA GTX 1080 GPU card yielded 10–13 ns a day. We analyzed all simulation trajectories using *MDTraj*^45^. We employed the *MDTraj* implementation of Baker–Hubbard method to compute the hydrogen bonds between residues. In our calculations, we used the following cutoffs to determine bound state: hydrogen–acceptor distance < 2.5 Å and donor–hydrogen–acceptor angle > 120°.

### Computing Kantorovich–Wasserstein distance

We computed ${d}_{KW}$ using the geomLoss python package^28^. For an all-atom trajectory, we first computed the center of mass positions for each bead in the six-bead model. The coarse-grained coordinates for each bead would resemble a point cloud in three-dimensional space and these point clouds were used as inputs to estimate ${d}_{KW}$. For instance, ${d}_{KW}$ for bead 1 between trajectories A and B was computed by providing the corresponding point clouds as input to the *Loss* function in the geomLoss package. A *Loss* function initialized to compute the *sinkhorn* distance with *blur* = *0.05* yields a good estimate of ${d}_{KW}$.

### Principal component analysis

For each of the IgG isotypes, we studied the joint essential dynamics of the six different trajectories using principal component analysis of their ${C}_{\alpha }$ atoms. We first aligned every frame in all the six trajectories to the Fc domain of the first frame in the 1HZH trajectory and extracted the positions of ${C}_{\alpha }$ atoms. In each trajectory frames 2 ns apart were chosen for the analysis. We used the position matrix to construct and solve the covariance matrix using the PCA module in the python scikit-learn library. We next projected the positions of the ${C}_{\alpha }$ atoms on to each principal vector and evaluated the contributions of each atom to the essential dynamics.

## Supplementary Information

Supplementary Figures.

## Data Availability

The datasets used and/or analyzed during the current study available from the corresponding author on reasonable request.
