# Supplementary material for: IgG1 and IgG4 antibodies sample initial structure dependent local conformational states and exhibit non-identical Fab dynamics
Source: Sci Rep. 2023 Mar 23;13:4791. doi: 10.1038/s41598-023-32067-9 (PMC10036467; doi:10.1038/s41598-023-32067-9)
Supplement: Supplementary file 1 — Supplementary Figures. [file 41598_2023_32067_MOESM1_ESM.docx]

**Supplementary Information for “IgG1 and IgG4 antibodies sample initial structure dependent local conformational states and exhibit non-identical Fab dynamics”**

Ramakrishnan Natesan^a^ and Neeraj J. Agrawal^a,*^

^a^Amgen Inc., Process Development, 360 Binney St, Cambridge, Massachusetts 02141, USA

^*^All correspondence should be addressed to

Neeraj J. Agrawal

Amgen Inc., Process Development, 360 Binney St, Cambridge, Massachusetts 02141, USA

Email: agrawaln@amgen.com

*Phone: +1 (617) 444-5503*

***
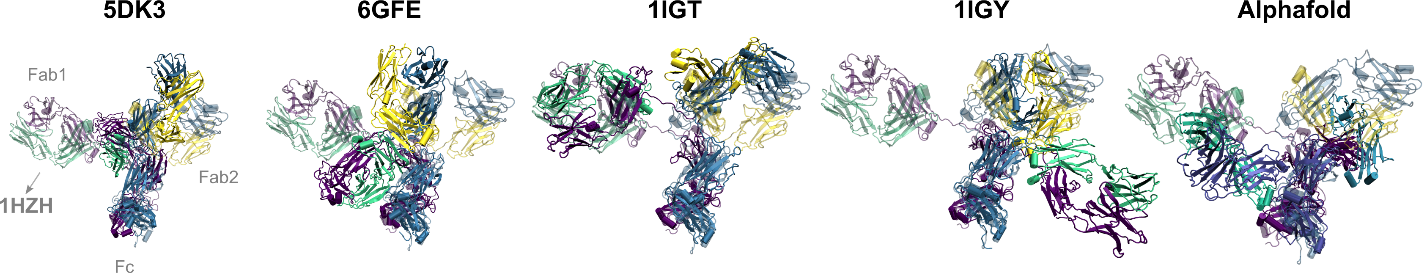
***

**Figure S1:** Snapshots of IgG4 molecules generated by homology modeling of 1HZH, 5DK3, 6GFE, 1IGT, 1IGY crystal structures, aligned to the Fc region of 1HZH. The reference 1HZH structure is shown in the background for comparison. The conformation of the AlphaFold structure post 1000 ns of relaxation is also shown.


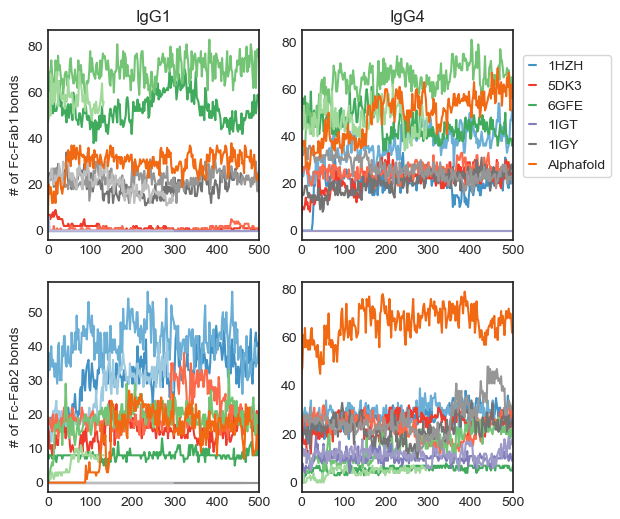


**Figure S2:** Timeseries of the number of non-covalent interactions in IgG1 and IgG4 molecules are shown in the left and right panels. The number of inter-domain interactions are shown for **(a)** Fc and Fab1 domains and **(b)** Fc and Fab2 domains for trajectories generated from five different crystal structures and from AI generated structures. Lines of the same colors denote data from multiple replicates.


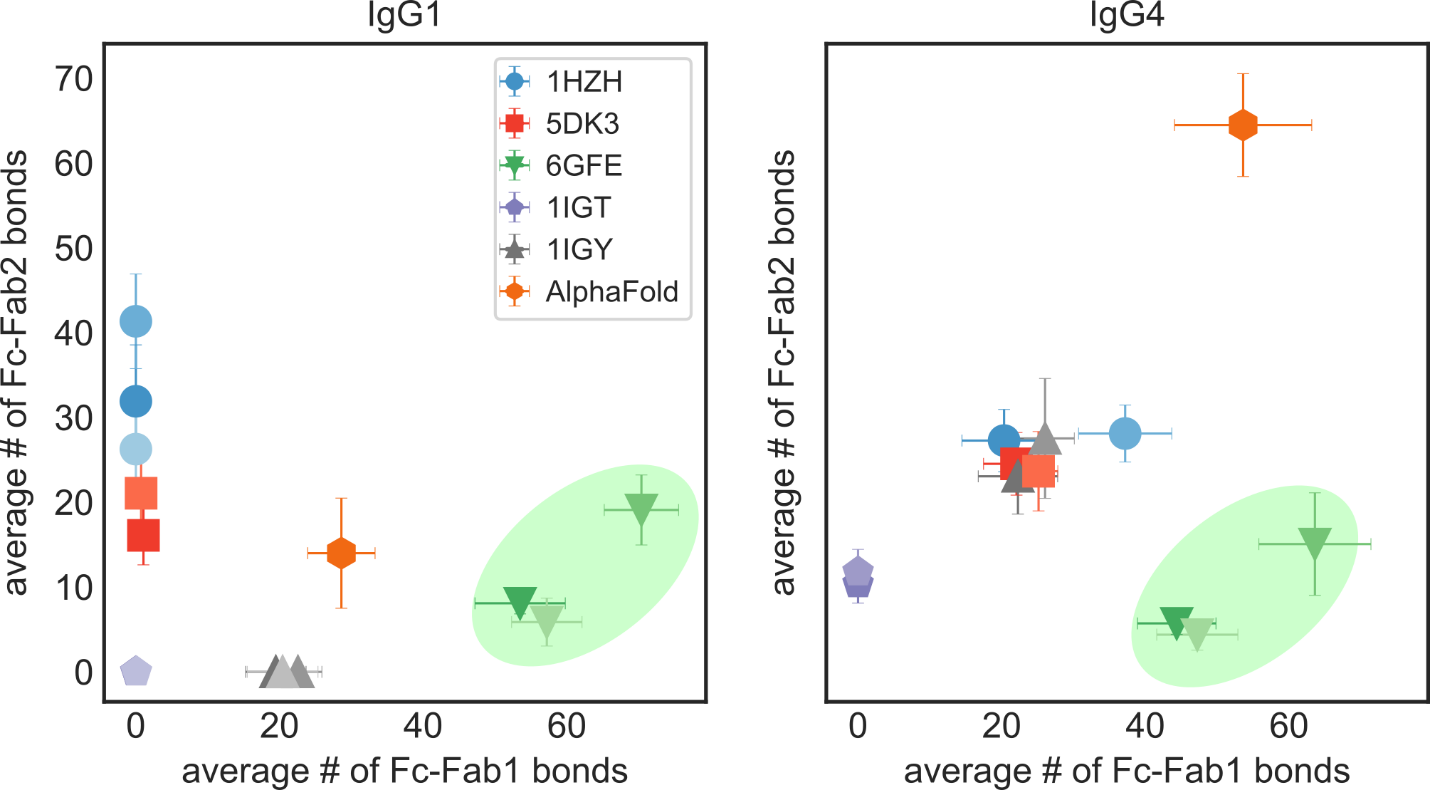


**Figure S3:** Scatter plots showing the number of Fc-Fab1 and Fc-Fab2 non-covalent interactions, averaged over the entire timeseries data shown in Figure S1. Data for IgG1 and IgG4 are shown in the left and right panels, respectively. The shaded region marks identical non-covalent interaction profiles in IgG1 and IgG4 for trajectories spawned from the 6GFE crystal structure


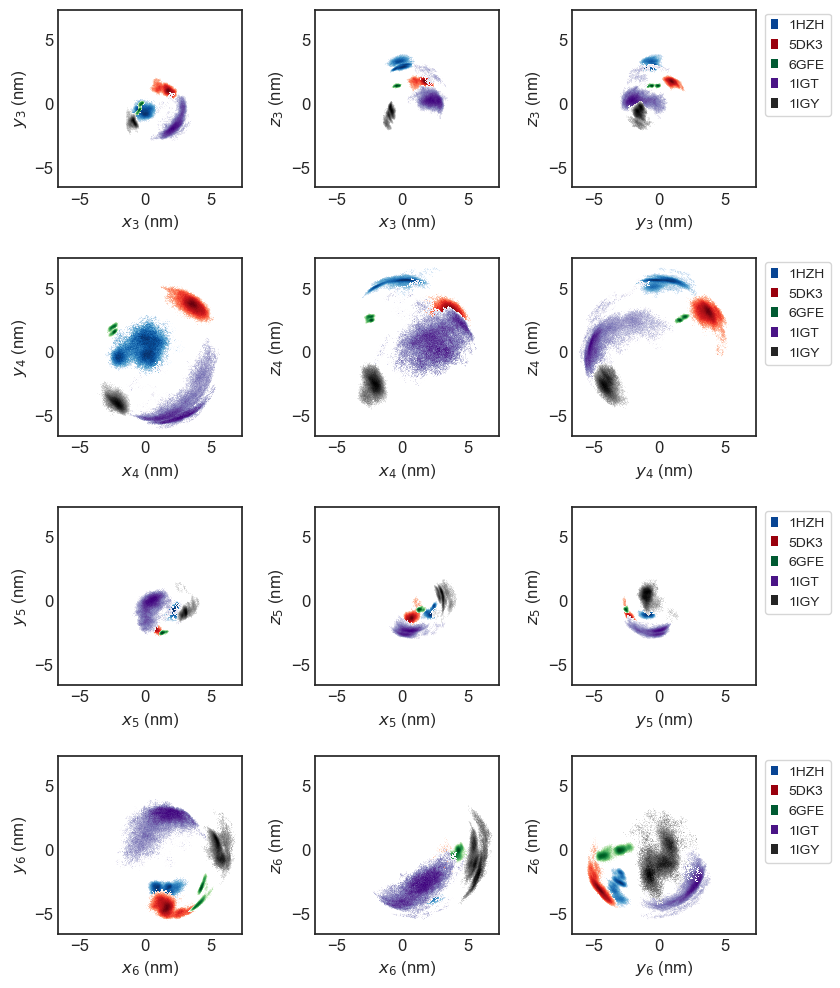


**Figure S4:** Projections of domain occupancy for IgG1 trajectories generated with five different crystal structures as the starting configuration. $x_{i}, y_{i}, z_{i}$ denote the x, y, z positions of coarse-grained bead $i$ with $i=3,4,5,6$.


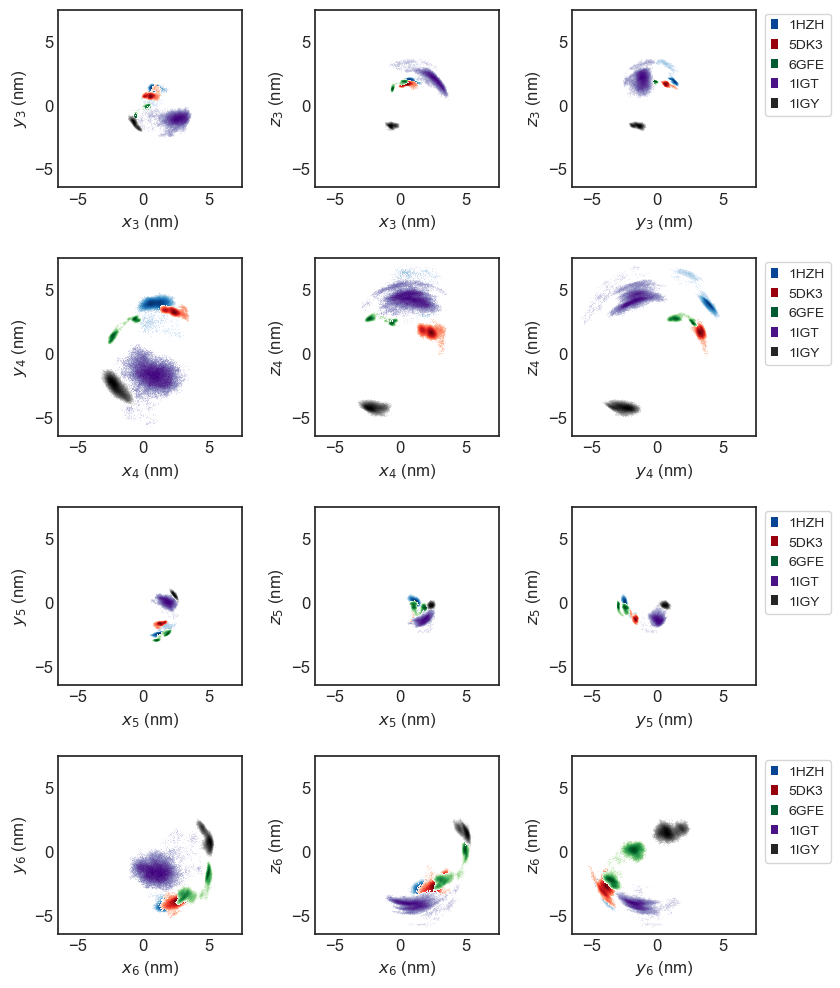


**Figure S5:** Projections of domain occupancy for IgG4 trajectories generated with five different crystal structures as the starting configuration. $x_{i}, y_{i}, z_{i}$ denote the x, y, z positions of coarse-grained bead $i$ with $i=3,4,5,6$.


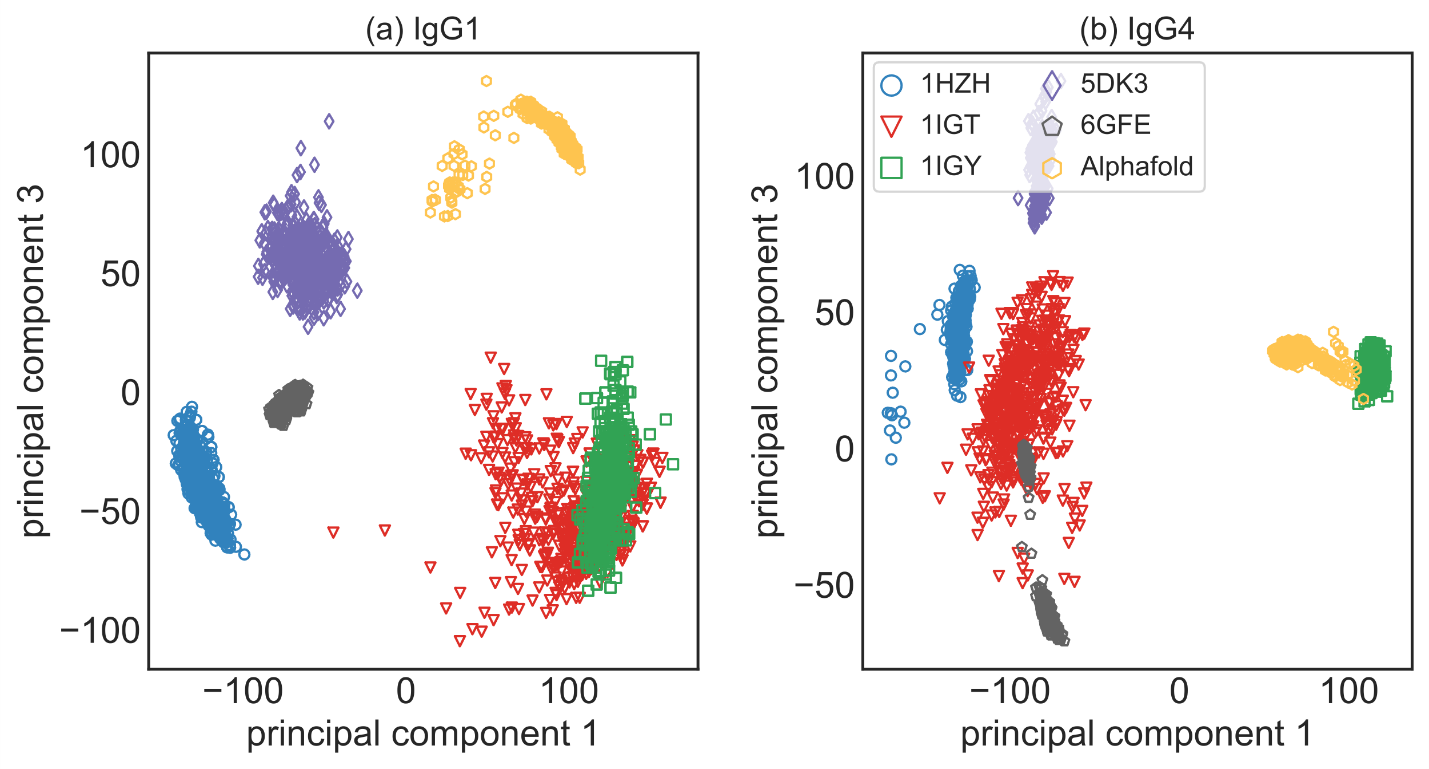


**Figure S6:** Projections of IgG1 and IgG4 trajectories along the first and third principal axes.


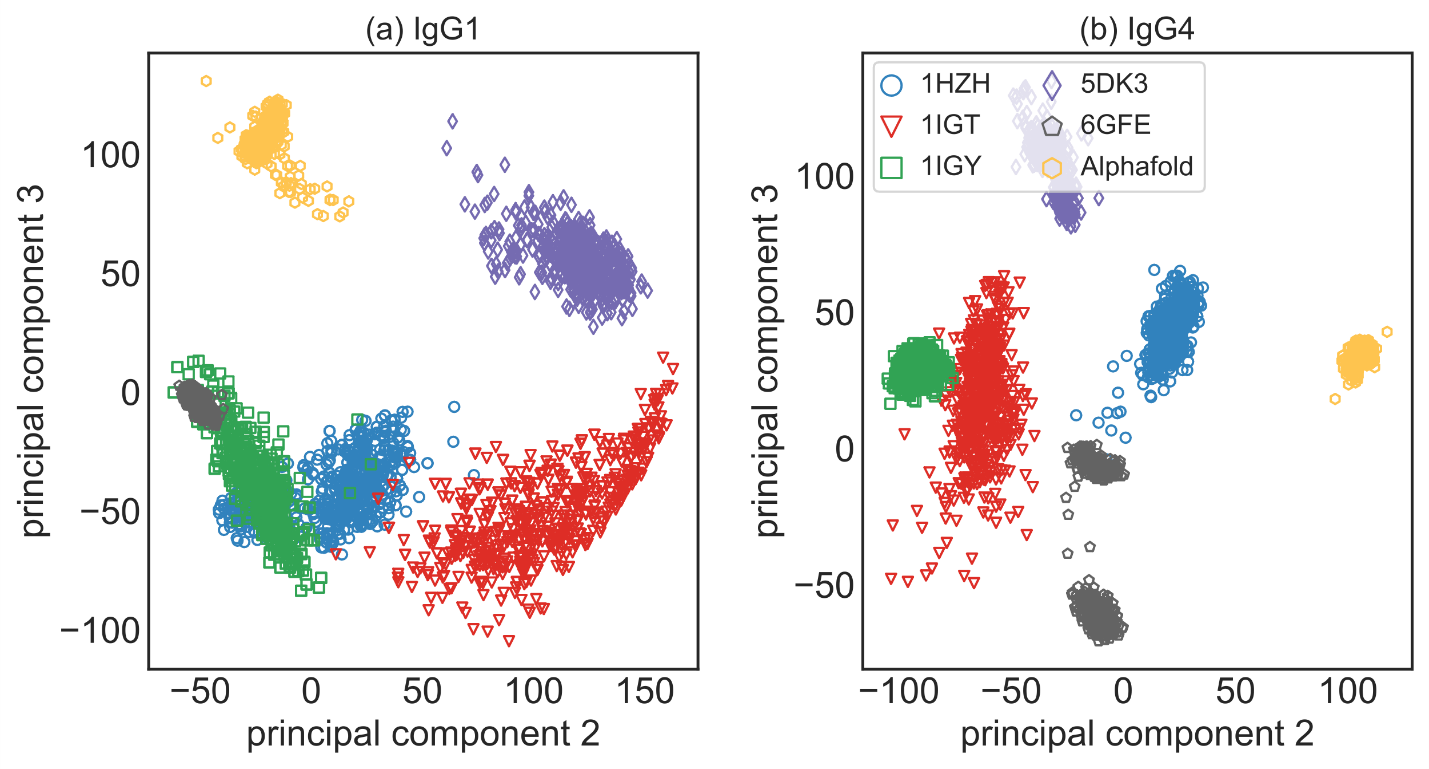


**Figure S7:** Projections of IgG1 and IgG4 trajectories along the second and third principal axes. Conformation states that appear to overlap along PC1 and PC2 (see Figure S12) show no such overlap along PC2 and PC3 indicating the conformational states accessed by the trajectories are mutually exclusive.


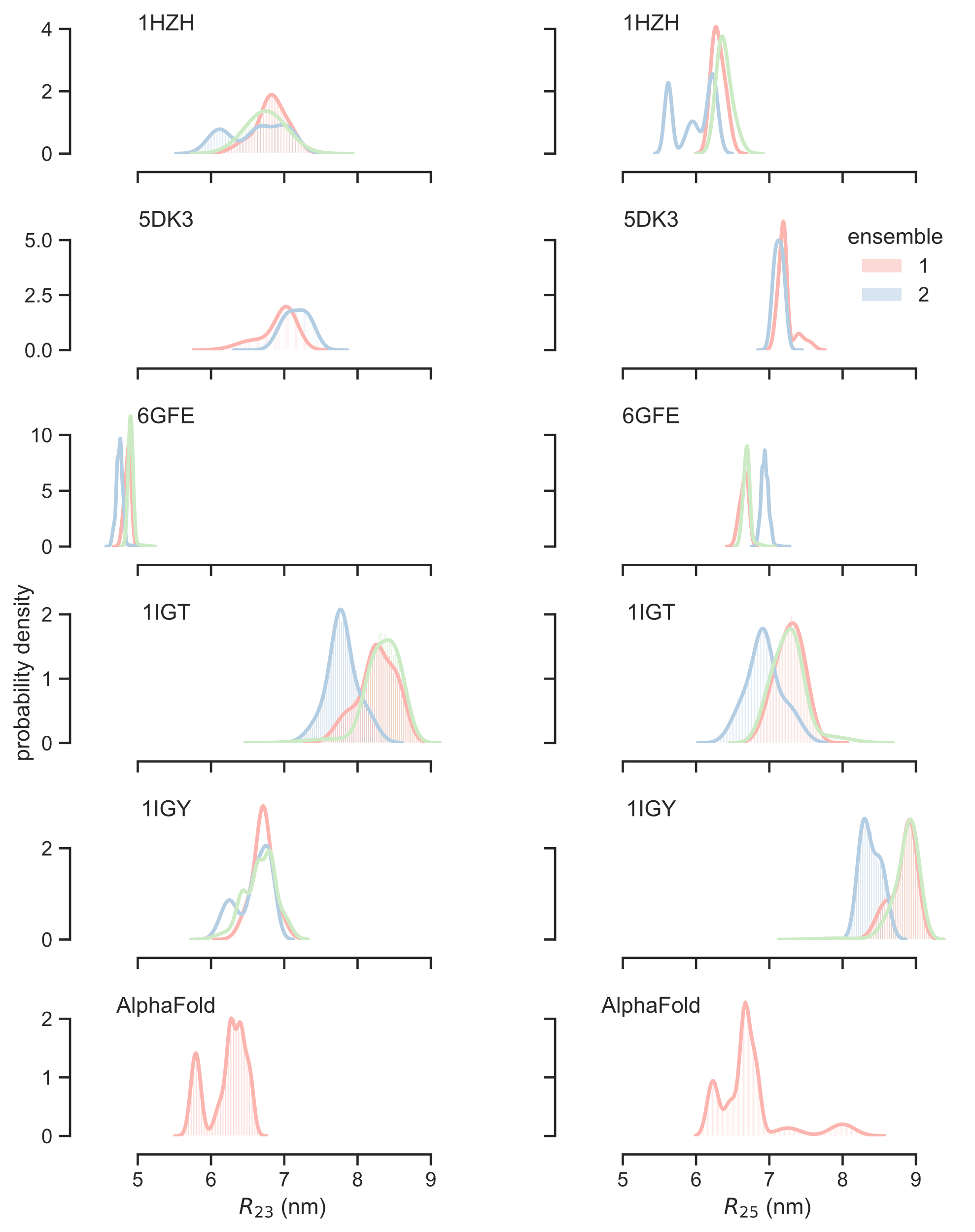


**Figure S8:** Probability densities of $R_{23}$ (left panels) and $R_{25}$ (right panels) for independent trajectories spawned from six different crystal structures of IgG1.


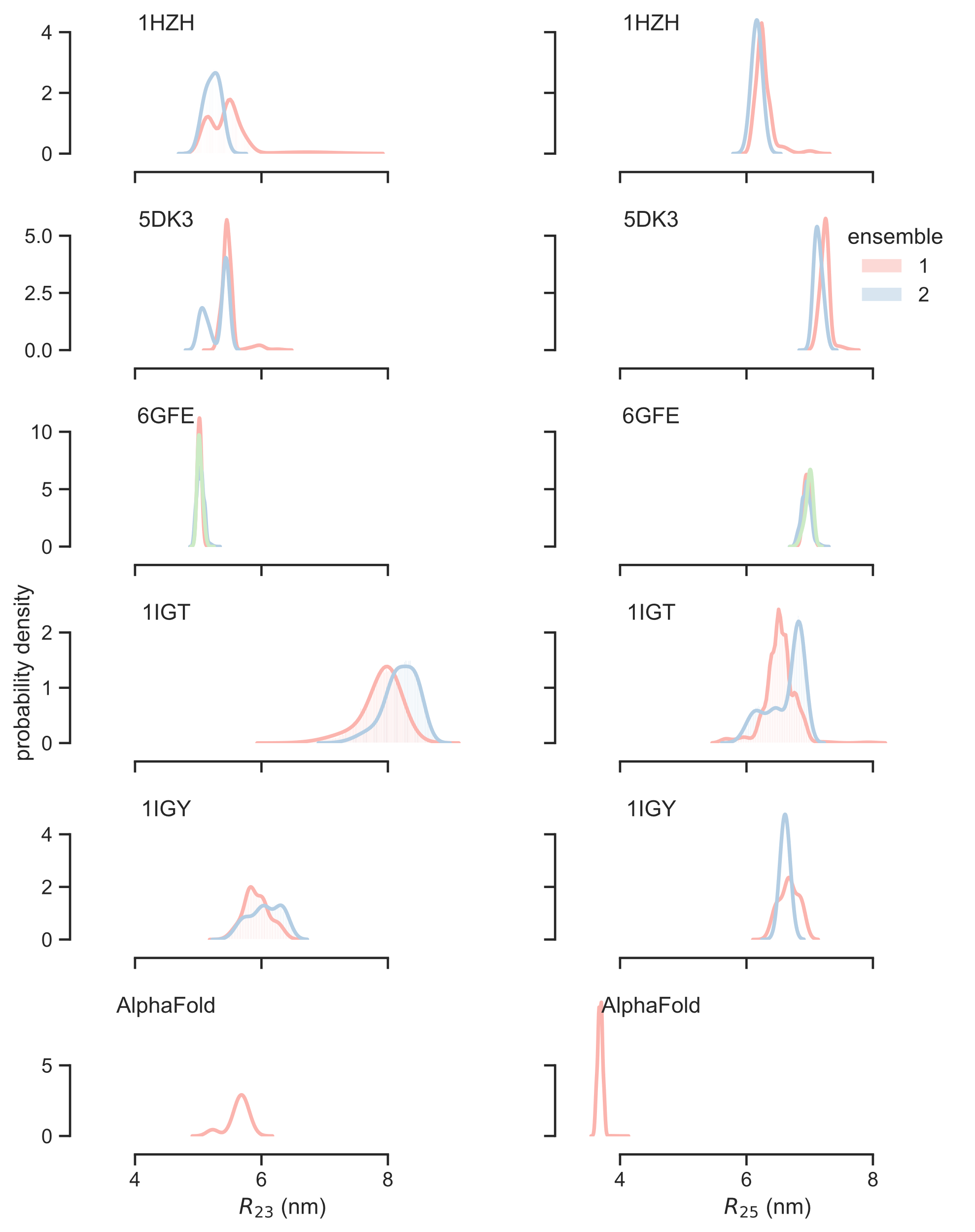


**Figure S9:** Probability densities of $R_{23}$ (left panels) and $R_{25}$ (right panels) for independent trajectories spawned from six different crystal structures of IgG4.


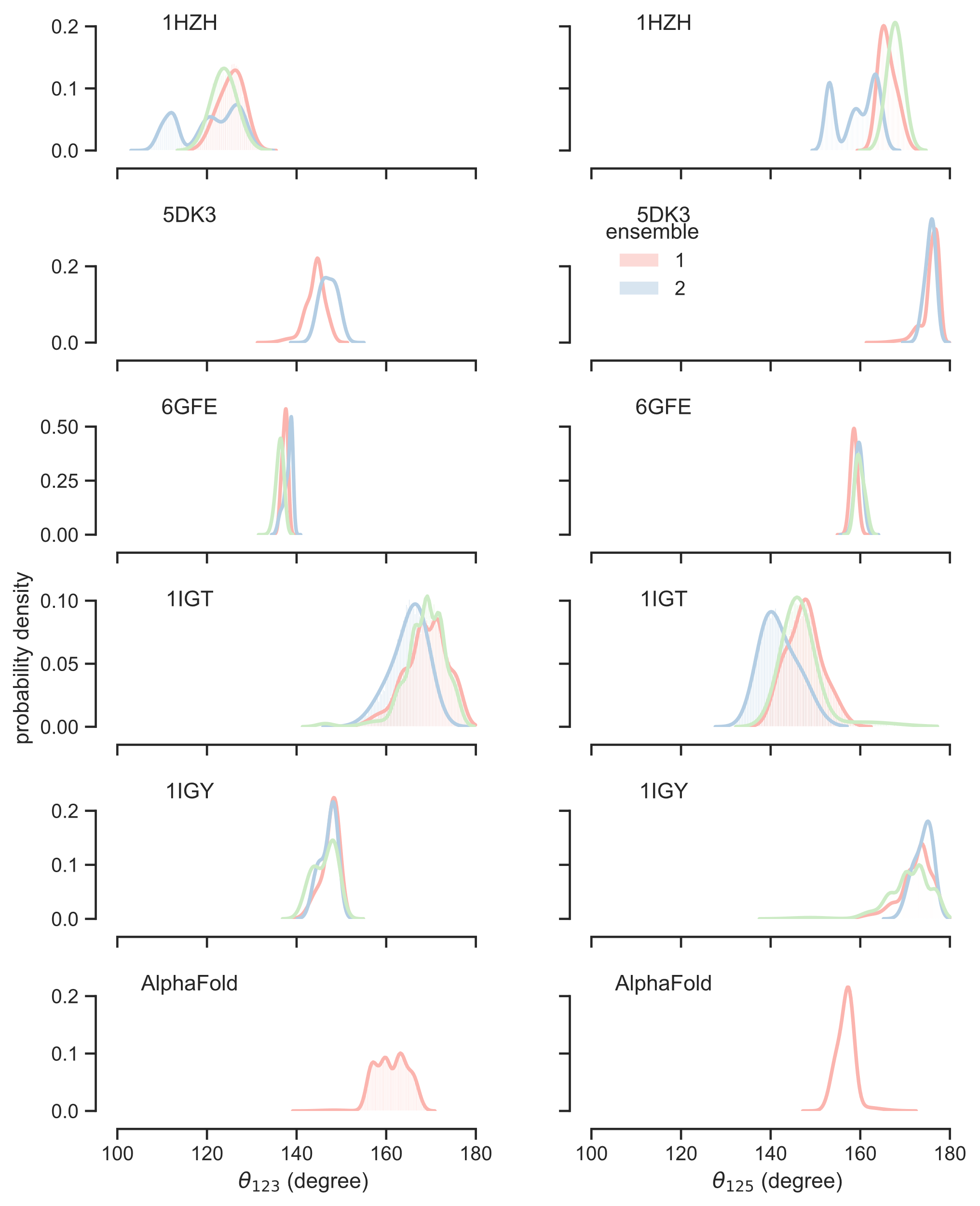


**Figure S10:** Probability densities of $\theta_{123}$ (left panels) and $\theta_{125}$ (right panels) for independent trajectories spawned from six different crystal structures of IgG1.


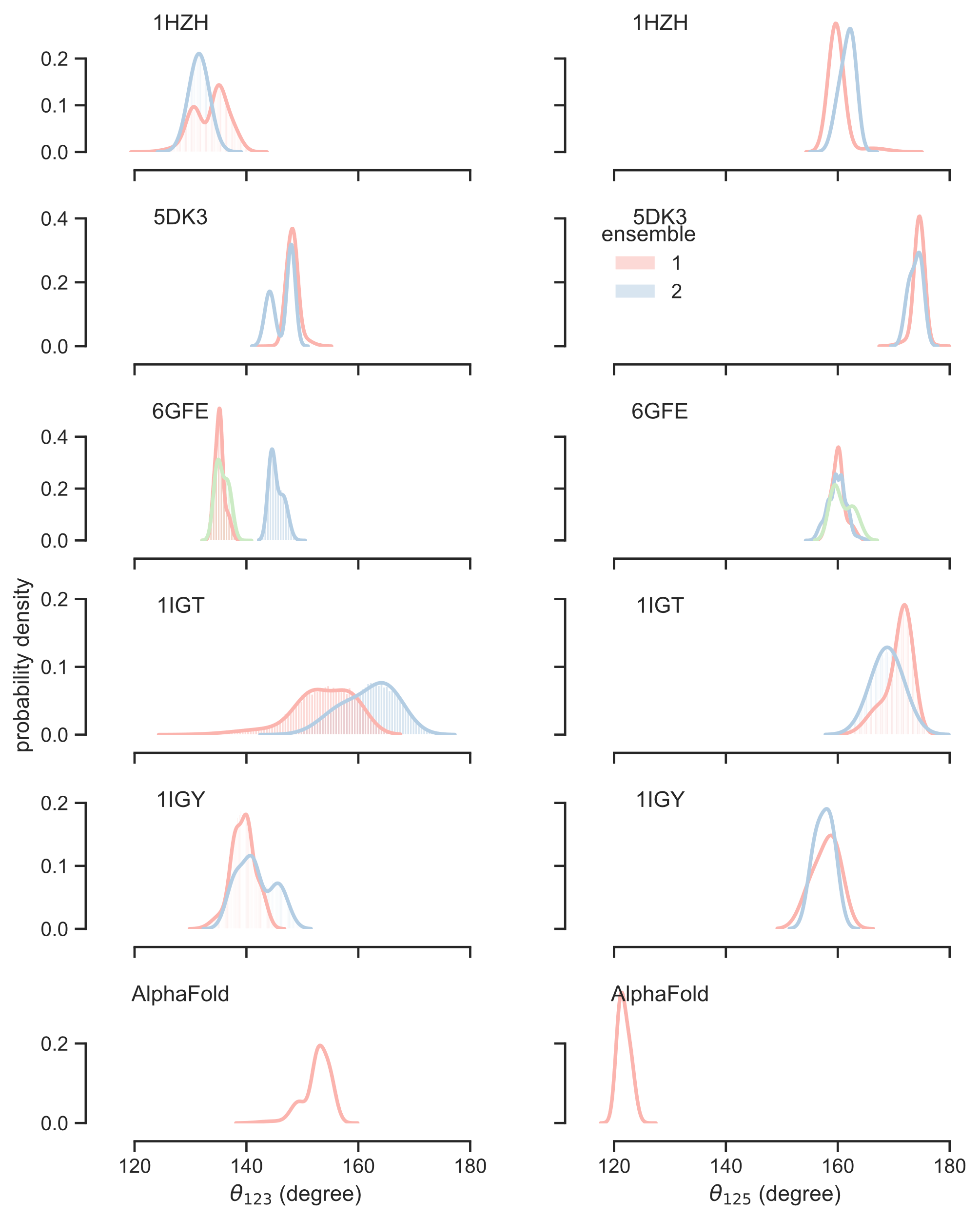


**Figure S11:** Probability densities of $\theta_{123}$ (left panels) and $\theta_{125}$ (right panels) for independent trajectories spawned from six different crystal structures of IgG4.


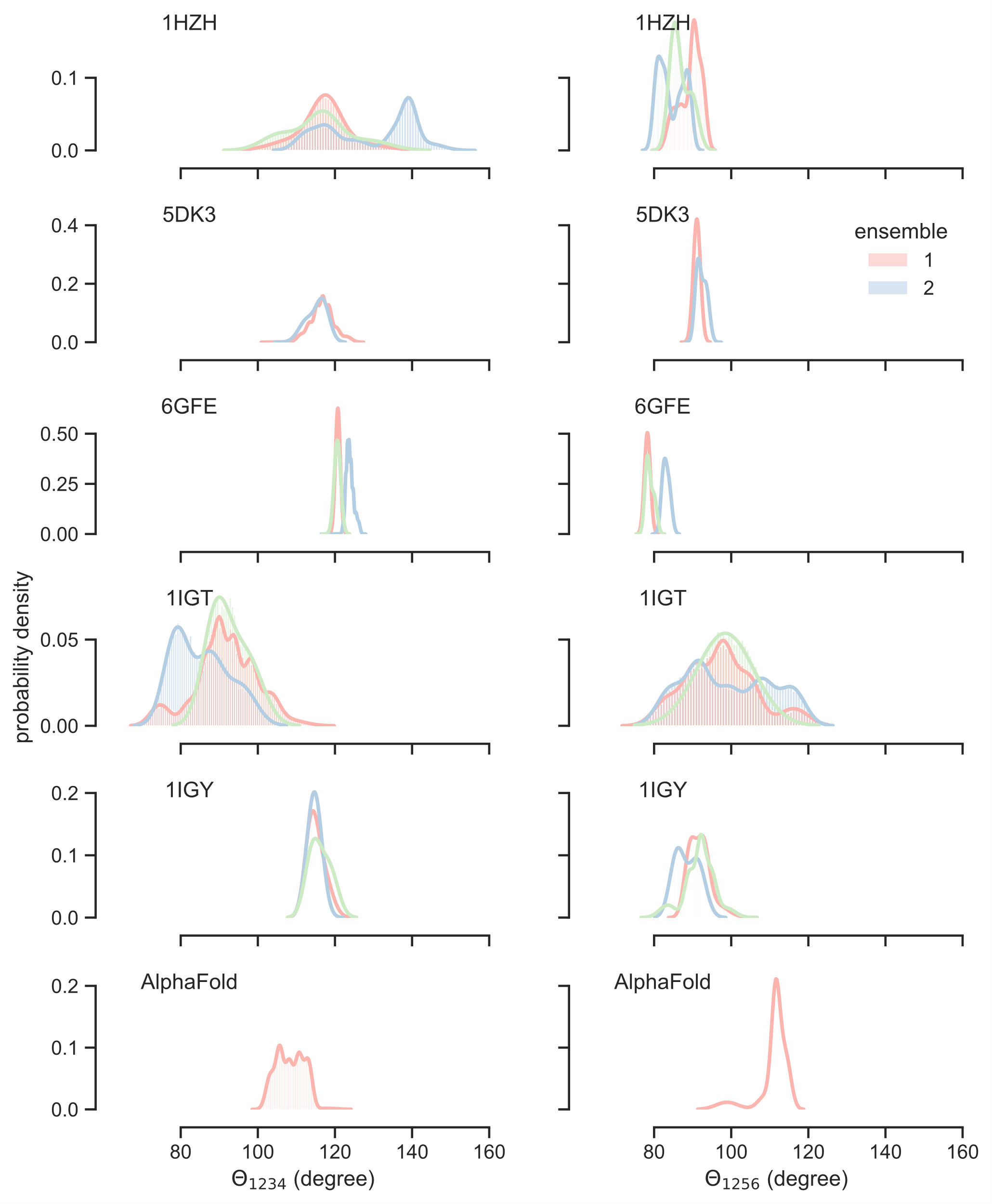


**Figure S12:** Probability densities of $\Theta_{1234}$ (left panels) and $\Theta_{1256}$ (right panels) for independent trajectories spawned from six different crystal structures of IgG1.


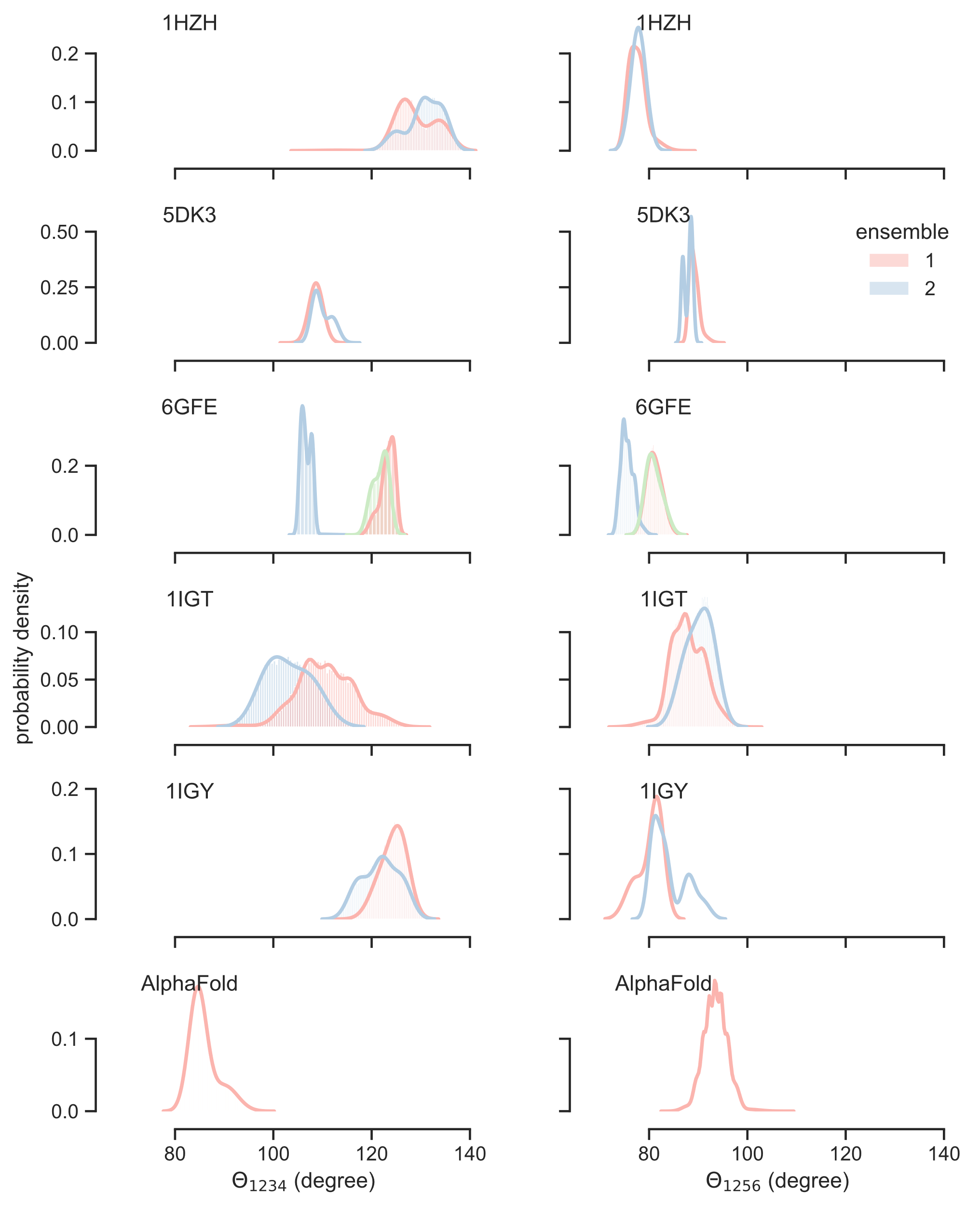


**Figure S13:** Probability densities of $\Theta_{1234}$ (left panels) and $\Theta_{1256}$ (right panels) for independent trajectories spawned from six different crystal structures of IgG4.
